# Early life nutritional programming of health and disease in The Gambia

**DOI:** 10.1017/S2040174415007199

**Published:** 2015-10-27

**Authors:** S. E. Moore

**Affiliations:** MRC Human Nutrition Research, Elsie Widdowson Laboratory, Cambridge, UK

**Keywords:** DOHaD, epigenetics, Gambia, immune programming, sub-Saharan Africa

## Abstract

Exposures during the early life (periconceptional, prenatal and early postnatal) period are increasingly recognized as playing an important role in the aetiology of chronic non-communicable diseases (NCD), including coronary heart disease, stroke, hypertension, Type 2 diabetes and osteoporosis. The ‘Developmental Origins of Health and Disease’ (DOHaD) hypothesis states that these disorders originate through unbalanced nutrition early in life and risk is highest when there is a ‘mismatch’ between the early- and later-life environments. Thus, the DOHaD hypothesis would predict highest risk in countries where an excess of infants are born with low birth weight and where there is a rapid transition to nutritional adequacy or excess in adulthood. Here, I will review data from work conducted in rural Gambia, West Africa. Using demographic data dating back to the 1940s, the follow-up of randomized controlled trials of nutritional supplementation in pregnancy and the ‘experiment of nature’ that seasonality in this region provides, we have investigated the DOHaD hypothesis in a population with high rates of maternal and infant under-nutrition, a high burden from infectious disease, and an emerging risk of NCDs.

## Maternal, infant and childhood nutrition and the Developmental Origins of Health and Disease (DOHaD)

Women, especially those who are pregnant or lactating, infants and young children are among the most nutritionally vulnerable groups, as a consequence of their physiologically higher nutrient requirements. In settings with high food insecurity, these additional nutrient requirements are often not met, resulting in an increased risk of adverse consequences. Under-nutrition in pregnancy leads to an increased risk of maternal morbidity and mortality and, for her infant, an increased risk of intrauterine growth retardation (IUGR) resulting in small for gestational age (SGA) infants, and also preterm delivery. Nutrition-related factors are estimated to account for about 45% of the deaths of children under 5 years of age globally.^1^ Among undernourished children who survive, many suffer from stunted growth, with associated deficits in a number of developmental outcomes including neurocognitive development.

In parallel to this widespread under-nutrition, many low- and middle-income countries (LMICs) are increasingly observing an epidemic of nutrition-related chronic diseases (NCDs) such as diabetes, cardiovascular disease (CVD) and cancers.^2^ While these problems have been traditionally thought of as mutually exclusive, the coexistence of under-nutrition and NCDs and the evidence that under-nutrition in early life may drive NCD risk across the life-course has changed our thinking. The DOHaD hypothesis argues that the early-life environment has lifelong effects on human health. Within the DOHaD research field, understanding the link between nutrition in early life (from pre-conception to early childhood) and life-long disease risk has been one of the key elements stimulating scientific interest. However, to date, the DOHaD concept has so far had little impact on national policies on maternal and infant nutrition.^3^ Generating an evidence base that combines both the immediate and long-term benefits of ‘optimal’ nutrition in pregnancy is critical.

## DOHaD in sub-Saharan Africa

To date, most data in support of the DOHaD hypothesis come from studies among high- and middle-income countries. However, the hypothesis may be especially pertinent among populations where high rates of early life under-nutrition are often followed by a later life transition to a lifestyle of adequate or excessive nutrition. In much of sub-Saharan Africa food insecurity threatens the lives of millions of vulnerable people, especially women, infants and young children. With more than a third of the population in sub-Saharan Africa currently classified as urban dwellers, and with this proportion predicted to increase, this rapid urbanization will also add a growing burden of NCDs to already stretched health services in these settings. In such settings, and within the DOHaD paradigm, there are three broad explanations linking the early-life environment to later disease risk. First, it is hypothesized that a severe challenge at a critical point of development, such as a frank deficiency of a critical micronutrient or exposure to maternal infection, may induce a permanent disruption to development. An extreme example is with frank deficiency of iodine during gestation, resulting in severe, non-reversible consequences to offspring.^4^ Second, if the early-life challenge is less severe, it may induce fetal adaptations, such as the slowing of fetal growth (and greater risk of the infant being born SGA). This concept of an adaptively growth-restricted organism has existed for many decades but was most clearly articulated in relation to DOHaD by Hales and Barker, who also coined the term ‘thrifty phenotype’.^5^ ^,^ ^6^ In the LMIC context, where the majority of SGA babies are born, the accepted thesis is that this early growth restriction would represent a successful adaptation so long as the nutritional circumstances in later life continue to match those that have induced the initial adaptation.^7^ In practice, however, this lifestyle is frequently not retained and many individuals who have experienced fetal and early-life nutritional hardships transfer to the urban areas and to a diet of relative energy excess with high intakes of fats and refined carbohydrates.^8^ ^,^ ^9^ It is this rapid transition within a single lifespan that is implicated by many researchers as a crucial causative agent in the explosion of non-communicable diseases in many such urban areas, and the basis of the thrifty phenotype hypothesis. The third explanation, and reflecting that the majority of environmental conditions lie within the normal range, are a set of adaptive responses that the embryo or fetus makes to these environmental cues that are not usually associated with reduced birth weight or indeed other adverse phenotypic consequences at birth.^10^ These processes, which have become termed as predictive adaptive responses, are made in expectation of the future postnatal environment. While some in-human evidence exists in support of the predictive adaptive response hypothesis in relation to the metabolic syndrome,^11^ understanding the mechanisms the underlie this apparent ‘mismatch’ is critical, and will be discussed later in this review.

On this backdrop, it is clear that any adverse consequence of a physiological adaptation to fetal/early infancy nutritional deprivation may be particularly important in resource-poor environments where gestational poverty is often followed by adult affluence. Research among populations with early-life nutritional deprivation but at risk of excess in later life is thus a critical but, I would argue, still neglected component of the DOHaD research field. In this paper I review our work with the DOHaD framework from rural Gambia, West Africa.

## DOHaD in The Gambia

Over the past three decades, evidence in support of the DOHaD hypothesis has come from studies using historical birth records, longitudinal birth cohorts and, more recently, the follow-up of randomized control trials. Our work in The Gambia has built on this model, benefiting from the availability of longitudinal data, prospective cohorts and trials of nutritional supplementation to assess the impact of early life under-nutrition on life-long health risk. Much of the early work in the field, led by Professor David Barker and colleagues from the MRC’s Environmental Epidemiology Unit in Southampton, UK, was based on their discovery of historical records from the now famous Hertfordshire, Preston and Sheffield cohorts. We have been able to contribute to the DOHaD field as a result of, first, the foresight of the late Professor Sir Ian McGregor, who established demographic data collection in rural Gambia over 60 years ago, and, second, an ‘experiment in nature’ resulting in annual patterns in a number of environmental determinants. This is reviewed below.

In October 1949, Dr McGregor joined the Nutrition Research cadre at the MRC’s Fajara station in The Gambia with the task to investigate the possible contributory role of parasitic infections on protein calorie malnutrition. Based on the incidence of splenic enlargement and anaemia in children under 10 years of age, coupled with its remoteness and a lack of medical services of any kind, he selected Keneba in West Kiang with the nearby villages for surveillance. Together with the two neighbouring villages of Manduar and Kantong Kunda, the villagers of Keneba were visited annually by Dr McGregor and his team to collect records of growth, morbidity and mortality on the population. In between these annual surveys, a system for routinely collecting data on all births and deaths in each village was established. In 1974, the Dunn Nutrition Unit in Cambridge, UK, under the directorship of Dr Roger Whitehead, took over the Keneba field station and, since this time, detailed records of nutrition and health have been collected by full-time resident field staff, in addition to the continued maintenance of the demographic records. The record keeping initiated by Sir McGregor in 1949 has been maintained continuously to date, and now forms the most enduring longitudinal demographic record of rural Africans.

The second feature that made The Gambia attractive for DOHaD research was the annual and pronounced pattern of seasonality observed in this environment. In the context of DOHaD, the salient environmental features are as follows: A subsistence farming existence is heavily influenced by a monomodal annual rainy season (July–October) which affects many aspects of diet, health and behaviour. A ‘hungry’ season, coinciding with the rains, occurs when the previous year’s food crops become depleted before the current year’s harvest. This is compounded by the need for hard agricultural labour, especially by women who are responsible for the rice harvest, and creates a marked negative energy balance (greatest in July–October) in all adults including pregnant women in whom monthly weight gain drops from 1500 g/month to only 400 g/month.^12^ Despite a demonstration of energy-sparing reductions in metabolism aimed at protecting fetal growth,^13^ ^,^ ^14^ a hungry season increase is observed in both SGA infants, and in preterm deliveries.^15^ The effect on birth weight is known to be primarily nutritionally mediated, since it is reversed by maternal dietary supplementation.^16^ Patterns of growth for Gambian infants are also influenced by the stage at which they enter the annual hungry season. When plotted from birth to 24 months of age and grouped by month of birth a ‘plaited string’ effect can be seen with individual curves crossing over each other in a complex way.^17^ This simply reflects the fact that during the annual hungry season growth is affected universally, but that the age when this occurs depends on month of birth. The annual seasonality also impacts on patterns of disease in rural Gambia, most notably in infants and young children. This seasonal variation of childhood diseases, including malaria and gastroenterological infections, has been noted since the first detailed community-based studies undertaken in Keneba in the 1950s,^18^ and its impact on growth has been well-documented since (e.g.^19^ ^,^ ^20^).

This seasonality creates a natural experiment in which month of birth acts as a strong proxy measure of fetal and early post-natal nutrition, and of certain maternal and infant infections and other environmental exposures. Together with the longitudinal demographic data this creates a model for exploring the impact of early-life exposures on long-term health outcomes among rural Gambians.

## Early-life malnutrition and cardio-metabolic health in rural Gambians

In the mid-1990s, we embarked on a series of studies in the Gambia, utilizing season of birth as a proxy indicator for early-life exposures, to explore the impact of the early-life environment on long-term health outcomes. In line with other programming work at the time, our work focused on CVD risk factors with an initial study designed to investigate whether fetal nutritional stress (using season of birth as a proxy measure for prenatal growth retardation) or early childhood malnutrition (using historical anthropometric records) predicted risk factors for CVD in adulthood. Subjects born during and immediately after the hungry season (July–December) were compared with those born in the harvest season (January–June). Early childhood malnutrition was defined using anthropometric and haematological status during infancy, extracted from the early records collected at the annual dry season surveys by Professor McGregor as described above. For each individual, the weight, height and haemoglobin measurements taken closest to the age of 18 months were used.

All adults with a known month of birth, born between 1949 and 1974 and still residing in the three villages were invited to participate. At the time of the study the mean age of the study participants was 35.8 years. A broad panel of CVD risk factors was assessed, including fasting plasma glucose, insulin and blood lipids. An oral glucose tolerance test (OGTT) was performed, and blood pressure and anthropometric measures collected.^21^

In this initial, small, but detailed observational study on young adults we demonstrated that, in rural Gambia, small babies, including those born during the nutritionally challenging hungry season, maintain excellent cardiovascular health into adulthood, with a complete absence of metabolic disease, so long as they retain their ‘lean, fit and frugal’ lifestyle in the rural areas.^21^ Furthermore, despite significant undernutrition in infancy, the risk factors for adult disease were not related to the subject’s nutritional status as children.

In 2011, 14 years after this first study, we traced this group of adults for a repeat study of metabolic health. Despite no direct contact during the intervening 14-year period, we were able to trace 80% of the original cohort of Gambian adults, for measurement of anthropometry and blood pressure, and a 120-min OGTT was repeated (S.E. Moore, unpublished data).

Table 1 summarizes the key characteristics of the study population we were able to follow up, with their data from 1997 and 2011 presented. As with the original study, there was a heavy bias of women within the sample (83% of total in 1997, 85% in 2011) this being a consequence of a difficulty in recruiting young adult men during the first phase of the study.^21^ Data for males and females have therefore been combined. In 1997, only subjects resident in West Kiang (rural) were recruited. In 2011, we traced subjects where possible to their current location within The Gambia. A total of 154 (88%) subjects remained in rural West Kiang, 19 (11%) lived in the urban ‘Kombos’ area, while the remaining two (1%) lived in the rural areas outside of West Kiang.

**Table 1:** Risk factors for cardiovascular disease among rural Gambian adults at baseline (1997) and follow up (2011)

	Mean (s.d.) or percentage^a^	
	1997	2011^b^
Age (years)	36.0 (7.76)	50.0 (7.77)
Weight (kg)	55.9 (9.18)	61.5 (13.2)
Height (cm)	161.2 (6.94)	163.7 (6.84)
BMI (kg/m^2^)	21.5 (3.08)	22.9 (4.50)***
Overweight/obese (%)	6.86/1.14	18.9/6.29***
Fat mass (kg)	na	18.1 (9.10)
Trunk fat mass (kg)	na	8.97 (4.87)
Systolic blood pressure (mmHg)	106.5 (10.5)	116.7 (17.5)***
Diastolic blood pressure (mmHg)	69.7 (7.68)	71.3 (10.2)***
Hypertensive (%)	13.1%	31.6%***
Glucose T0^c^ (mmol/l)	5.31 (5.23, 5.38)	5.30 (5.19, 5.43)
Glucose T30 (mmol/l)	7.41 (7.22, 7.61)	7.36 (7.07, 7.67)
Glucose T120 (mmol/l)	5.05 (5.28, 6.15)	5.46 (5.20, 5.74)**
Insulin T0 (pg/ml)^d^	4.05 (4.03, 5.75)	5.98 (5.46, 6.55)***
Insulin T30(pg/ml)	39.7 (36.6, 43.1)	48.2 (43.2, 53.8)***
Insulin T120 (pg/ml)	13.2 (11.6, 15.0)	20.2 (18.0, 22.7)***
Impaired glucose tolerance (%)	0.57	7.43***
Type 2 diabetes mellitus (%)	0	2.86*

Data presented for the 175 individuals seen in 1997, who were traced in 2011.

a Geometric means (95% CIs) have been presented for skewed variables

b Paired T-tests (continuous data) or χ^2^ (prevalence data) used to test for differences between timepoints. *P<0.05, **P<0.01, ***P<0.001

c T0 – Baseline (fasting); T30–30 minutes post-glucose load; T120–120 minutes post-glucose load.

d Insulin data from 1997 originally measured in pmol/l, converted by a factor of 6.945 to be presented in pg/ml.

At follow-up, 7.4% of the subjects had impaired glucose tolerance (IGT; 2 h plasma glucose level >7.78–11.0 mmol/l^22^) and 5 (2.9%) had a provisional diagnosis of Type 2 diabetes mellitus (2 h glucose ⩾11.1 mmol/l). This is considerably higher than in 1997, when, and using the same criteria, only 1% of the 175 subjects currently remaining in the cohort had a diagnosis of IGT, and none were classified as having Type 2 diabetes mellitus (Table 1). Incidence of hypertension increased from 12% in 1997 to 31% in 2011, while the prevalence of overweight (BMI⩾25 kg/m^2^) and obesity (BMI⩾30 kg/m^2^) increased from 6.8% and 1.14% respectively in 1997 to 18.9% and 6.3% respectively in 2011. Consistent with the original study in 1997, no associations were observed between markers of the early-life environment and adult measures of CVD risk (S.E. Moore, unpublished data). Whether the observed increases in CVD risk reflect secular trends, or the effect of ageing alone, is difficult to assess but highlights that, with the ongoing nutrition transition, future studies investigating the DOHaD paradigm in relation to CVD risk may be warranted.

## Early-life malnutrition and survival in rural Gambia

**Fig. 1:**
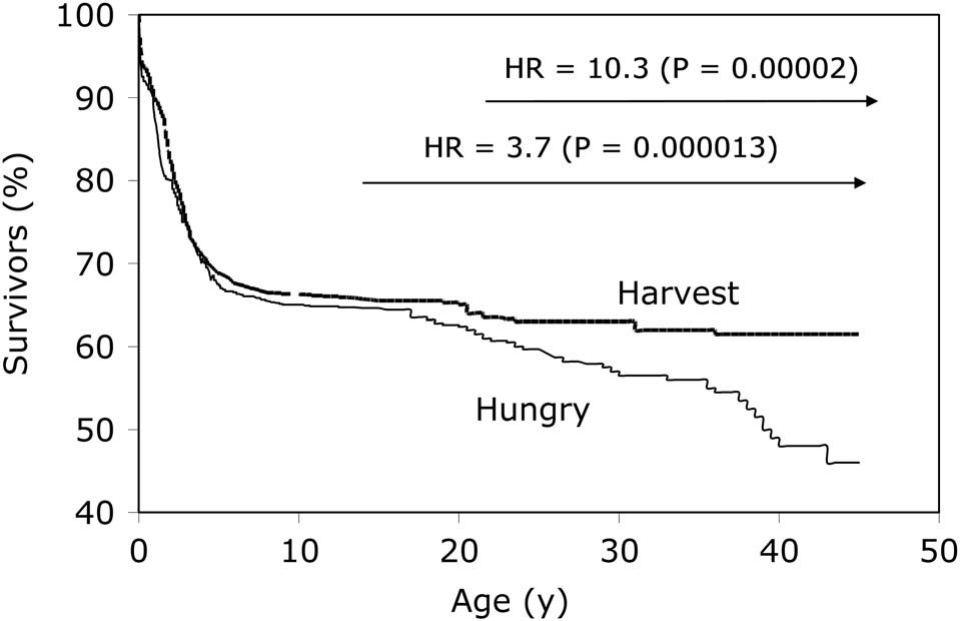
Kaplan–Meier survival plots by season of birth. Adapted from Moore *et al*. Thin line represents ‘hungry season’ (July–December) births; thick line represents ‘harvest season’ (January–June) births.

Our interest in exploring the DOHaD hypothesis within the Gambian context took a directional change in the later 1990s, owing to an almost serendipitous finding within our data. The ability to use seasonality as a proxy measure for early-life environmental exposures, alongside 5 decades of demographic data, presented an opportunity to look at survival according to season of birth. What we found was unexpected: birth during the annual hungry season (July–December) led to a significantly greater risk of premature adult mortality compared with birth during the annual harvest season (January–June).^23^ Relative to the harvest season, the hazard ratio for early death in hungry-season births rose from 3.7 for deaths over the age of 14.5 years (*P*=0.000013) to 10.3 for deaths over the age of 25 years (*P*=0.00002) (Fig. 1). Cause of death was ascertained for over 75% of cases from either field clinic records, hospital records or verbal autopsy from relatives for over 75% of cases, revealing that the majority of deaths for which cause was known had a definite or possible infectious aetiology; none were from non-communicable diseases.^24^ A case–control analysis comparing nutritional status of the cases (premature adult deaths from both seasons) against controls matched for sex and year of birth, and randomly selected from the database, demonstrated that anthropometric and haematological status at 18 months of age was almost identical in cases and controls, indicating an earlier origin of the insult (i.e. occurring pre- or early post-natally).

This finding that susceptibility to premature death in young adulthood from primarily infection-related causes, was strongly related to early life events, predicted by season of birth, suggested that one or more components of human immune function have been permanently damaged by early-life exposures. The finding in a setting of pronounced seasonality in many aspects of diet, nutritional status, behaviour and disease makes identification of the initial aetiological insult particularly challenging. However, we have argued elsewhere^24^ that – in this environment – the most likely explanation for any long-term programming effect on the immune system is likely to be nutritional in origin, and our recent research has focused on elucidating the mechanisms linking the early life nutritional environment to long-term defects in functional immunity.

## Early-life nutritional programming of the human immune system

Epidemiological data to support an early life effect on immune development primarily comes from evidence linking IUGR to an increased risk of both morbidity and mortality from infectious diseases.^25^ ^,^ ^26^ Many components of the human immune system develop early during fetal life and by birth all systems are in place to allow the rapid maturity of the adaptive immune system towards immune competence. Although the immune system is qualitatively complete at birth, exposures during early life are critical for driving the adaptive expansion of the immune system. A developmental insult leading to the breakdown of any one of the complex pathways required to protect the host against pathogenic organisms could lead to an increased susceptibility to infectious disease. Following our observation of increased mortality among hungry season births, we established a programme of work to test the hypothesis that enhanced nutrition early in life promotes infant immune development, translating into improvements in later functional outcomes. Below I outline some of the key studies from this work in The Gambia.

Much of our initial work focused on the thymus as a potential target for early nutritional programming. The rationale for this was three-fold: First, the thymus is central for the early development of controlled adaptive immunity, with thymic development known to start early in the first trimester of pregnancy; second, thymic development is disproportionately affected by both maternal and infant undernutrition, with the description of the thymus as a ‘barometer of malnutrition’ coming long before any role for the thymus gland in immunological memory was known;^27^ and, third, post-natal development of the thymus can be assessed sonographically using a non-invasive validated method in which the transverse diameter of the thymus and the saggital area of its largest lobe are multiplied to give a volume related thymic index (TI).^28^ ^,^ ^29^ Studies using rodent models have shown that low birth weight, resulting from maternal protein restriction during pregnancy, followed by post-natal catch up growth is associated with shortened lifespan, whereas protein restriction and slow growth during lactation leads to an increased lifespan.^30^ It is proposed that these effects are mediated through differential thymic growth.^31^ Data from infants in Guinea-Bissau, West Africa, and our own data from Bangladesh, indicate that a small thymus in infancy is an independent risk factor for infection-related mortality in infancy.^32^ ^–^ ^34^ Such data support the importance of early-life thymic development for life-long health.

In a prospective birth cohort study of 138 Gambian infants, we demonstrated that sonographically assessed TI varies significantly with season of birth and subsequently (and more strongly) with season of measurement.^35^ Characteristic tracking of an individual’s TI (even after adjustment for body size) was observed, consistent with a possible role for the thymus in long-term programming. A seasonal influence was also observed in lymphocyte subpopulation counts from the same cohort,^36^ indicating a corresponding disruption to T-cell numbers and consistent with the hypothesis that permanently programmed defects in thymic function may affect adult cell-mediated immunity.

Of particular interest was the observation that the seasonal effect on TI was greatest when the infant was 8 weeks of age. Since, in this community, infants at this age are exclusively breast fed, are growing well and have minimal incidence of active infections, this observation could suggest the involvement of breast milk factors on thymic development. Using breast milk samples collected at the same time that TI was measured, Ngom *et al.* tested for the presence of a candidate breast milk immune factor interleukin (IL)-7; a cytokine critical for thymic and T-cell development. Despite considerable monthly variation, breast milk IL-7 concentrations were significantly lower in the hungry/wet season compared with the harvest/dry season, suggesting a putative role for breast milk factors in thymic development.^37^

Follow up of a sub-group of 472 children aged 6–9 years born during a trial of maternal nutritional supplementation found no consistent evidence for linkage between various *in vivo* functional measures of immune function and season of birth, size at birth or maternal pre-natal dietary supplementation.^38^ These results suggest that programmed deficits are more likely to relate to immunological memory than to initial responses suggesting that one hypothesis to explain the link between early-life exposures and later morbidity and mortality is that the observed immuno-incompetence results from an underlying defect in immunological memory, similar to the immuno-senescence of old age. To test this hypothesis, in a study of 60 Gambian men (18–23 years of age) selected to represent births in the hungry/wet *v*. harvest/dry season, we looked at markers of thymic function and T-cell parameters.^39^ ^,^ ^40^ CD4+ and CD3+ but not CD8+ counts were lower for those born in the hungry/wet season and CD8+ telomere length also tended to be shorter, possibly suggesting an elevated risk of premature immunosenescence in this population exposed to likely early under nutrition.^39^ ^,^ ^40^ Such effects could also be reflected in the phenotype and kinetics of T-lymphocytes in subjects. To investigate this hypothesis, Ghattas *et al.* ^41^ ^,^ ^42^ measured *in vivo* T-cell kinetics by stable isotope labelling of T-cell subsets combined with gas chromatography-mass spectrometry, T-cell phenotype (CD3, CD4, CD8, CD45RA and CD45RO expression), and frequency of single-joint T-cell receptor excision circles in young Gambian men. No differences were observed in T-lymphocyte homeostasis in relation to markers of perinatal nutritional compromise (birth season and birth weight).^41^ However, it may be that differences in functional immuno-responsiveness would become apparent in response to an immunological challenge.

To date, in summary, we have demonstrated: (a) that seasonal/nutritional effects on immune outcomes can readily be detected in the first year of life in Gambian infants,^35^ a finding also replicated among a larger contemporary cohort of Bangladeshi infants,^43^ appear undetectable in later childhood,^38^ but again detectable in later life;^40^ ^,^ ^44^ (b) that the detectable defects (thymic contraction, deviations in markers of thymic function^35^ ^–^ ^37^) all appear to be related to adaptive immunity; and, importantly, (c) that there is strong evidence of pre-natal influences given that differences in size adjusted thymic index are detectable at 1 day and 7 day post-partum.^35^ ^,^ ^43^ Attempts to relate these changes to specific nutrient deficiencies (e.g. vitamins A and C, zinc and protein-energy status) have so far not yielded positive findings. Our recent work, therefore, has focused on a proof-of principle intervention trial employing comprehensive multiple micronutrient (MMN) and protein-energy (PE) supplements.^45^ The Early Nutrition and Immune Development (ENID) Trial (ISRCTN49285450) is a randomized, partially blind trial to assess whether nutritional supplementation to pregnant women (from <20 weeks of gestation to term) and their infants (from 6 to 12 months of age) can enhance infant immune development. In pregnancy, women were randomized to four intervention groups (iron-folate, MMNs, PE, PE+MMN) and, from 6 months of age, infants are further randomized to a lipid-based nutritional supplement with or without additional MMNs. The primary outcome measures of the ENID Trial are thymic development during infancy, and antibody response to vaccination. At the time of writing this review, analysis of the primary outcome data from the ENID Trial was ongoing, and results will be reported subsequently.

Linking our research between NCD risk and immune function in this environment, we have also explored the early-life predictors of chronic inflammation. Risk for a number of NCD outcomes has been shown to be strongly predicted by an individual’s habitual levels of low-grade inflammation, which can be measured using blood markers such as C-reactive protein. However, very little is known about the origins and determinants of these markers. In a study of 320 Gambian adults (born in West Kiang, but traced to their current residence across The Gambia) we measured fasting levels of a comprehensive panel of eight inflammatory markers (C-reactive protein, serum amyloid A, orosomucoid, fibrinogen, α 1-antichymotrypsin, sialic acid, interleukin-6 and neopterin) in relation to early life measures. Despite the inclusion of a range of early life measures, in particular measures of early postnatal growth, and a large and varied number of markers to assess systemic inflammation, little evidence was found for an effect of early life on later inflammatory status.^46^

## Follow up of randomized control trials

If maternal nutrition is an important cause of developmental outcomes, better outcomes would be expected in children whose mothers were supplemented in pregnancy. In rural Gambia, work from the MRC has previously demonstrated that supplementation during pregnancy with protein and energy dense biscuits during gestation, results in significantly improved birth weight among offspring: Infants whose mothers received the supplement during pregnancy were on average 136 g heavier than control infants (whose mothers had received the supplement during lactation).^16^ When stratified by season, birth weight increased by 201 g in the hungry season (June–October) and by 94 g in the harvest season (November–May). These infants were born between 1989 and 1994, and have been followed up through childhood and into adolescence as part of our DOHaD programme of work, with outcomes including immune function,^38^ metabolic risk^47^ ^,^ ^48^ and cognitive function.^49^ However, despite a significant impact on birth outcomes of this intervention, no consistent impact has been observed with long-term health outcomes. There are a number of possible explanations for this lack of effect including age at follow-up (too young to observe differences in outcome measured, specifically in markers of immune-senescence), choice of outcome measure (not appropriately sensitive), low risk of disease incidence (especially in the case of CVD outcomes) or loss of ‘cases’ to follow-up in this setting where rates of neonatal and early-infant mortality remain high.^50^ However, it is increasingly becoming recognized that the processes linking early-life exposures to later disease risk may not manifest through fetal growth, and hence birth weight, and thus interventions in mid- to late-gestation, while impacting on birth outcomes, may not be relevant in terms of disease programming. The follow up of trials of maternal supplementation designed specifically to assess the impact on longer-term health outcomes are warranted.

## Season of conception and epigenetic regulation of disease in The Gambia

An important challenge to all of our work in The Gambia, as with work within the broader DOHaD field of research, has been to identify the underlying mechanisms. Epigenetic regulation has emerged as a strong candidate to explain the link between exposures in early life and later disease risk. Epigenetic modifications of DNA convey stable alterations in gene expression, not mediated by changes in DNA sequence, and errors in epigenetic processes are heavily implicated in numerous developmental defects and diseases. In contrast to genetic traits, epigenetic traits are modifiable by external exposures, particularly diet. Understanding the impact of maternal diet on the epigenome has thus become a critical component of the DOHaD field of research, and our own work in The Gambia is contributing to this understanding.

DNA methylation, which involves the addition of methyl groups (-CH_3_) to cytosine-phosphate-guanine dinucleotides, appears to be the most stable epigenetic mark and, when established during early ontogeny, can persist for life.^51^ Biological methylation is dependent on methyl group donors and cofactors supplied by diet and, therefore, dietary deficiency at critical periods of epigenetic ‘reprogramming’ is hypothesized to be a mechanistic link between diet and life-course disease risk.^52^ Metastable epialleles (MEs) are genomic regions at which DNA methylation is established stochastically in the early embryo then stably maintained in differentiated tissues, leading to inter-individual epigenetic variation that affects multiple cell types.^53^ In an initial study, Waterland *et al.* demonstrated that season of conception significantly influences the methylation of candidate MEs in children.^54^ Contrary to the initial hypothesis, DNA methylation was highest in individuals conceived during the annual wet/hungry season. Recent work from our group has further exploited the seasonal model in rural Gambia to demonstrate that maternal nutritional status at the time of conception affects epigenetic changes at MEs in her infant.^55^

In a preliminary, observational study of diet intake and nutritional status of Gambian women across a complete calendar year, we demonstrated a pronounced seasonal variation in a number of key methyl-donor biomarkers.^56^ Of key interest, and in line with the previous observation of greater methylation in individuals when conceived in the annual wet season, was the observation of a higher concentration of a number of key biomarkers (including folate, riboflavin and betaine) over the wet/hungry season, compared to the dry/harvest season. This observation likely reflects the need for greater dietary diversity (increased consumption of ‘bush’ foods) at this time of the year, when home food supplies from the previous harvest become depleted. In a prospective study of pregnant women and their infants, selected to represent conceptions at the ‘best’ (February–April) and ‘worst’ (July–September) times of the year, we then went on to show that biomarkers of 1-carbon metabolism measured in maternal blood samples collected close to conception correlated with DNA methylation measured in infant blood and hair follicle DNA samples.^55^ Deficiency in vitamins B2 (riboflavin) and B6, and aberrant increases in intermediary markers of 1-carbon metabolism [especially homocysteine and S-adenosylhomocysteine: S-adenosylmethionine (SAM:SAH) ratio] were significant predictors of impaired methylation, indicating a putative link between maternal supply of 1-carbon metabolites and methylation in very early embryonic/fetal life.

More recently, and using two-independent genome-wide approaches to assess DNA methylation among this group of Gambian infants, we identified the genomically imprinted *VTRNA2-1* as the epiallele most sensitive to season of conception effects.^57^ VTRNA2-1 is a putative tumor suppressor and modulator of innate immunity, and thus this finding constitutes a possible pathway linking the early environment to later human disease, via epigenetic alteration. Ongoing work by our group in The Gambia is investigating this association between maternal nutritional status around the time of conception and epigenetic programming in more detail.

## Summary

Our work in rural Gambia within the DOHaD field of research was predicated on the availability of long-term detailed health and demographic records, on the backdrop of an ‘experiment of nature’ created by a pronounced annual seasonal pattern. We initially aimed to contribute to the general DOHaD field, providing evidence for the early-life programming of cardio-metabolic health. Instead, we have provided evidence that communicable disease risk may also have origins during the early-life period and that this may be epigenetically programmed. Future research will follow these observations, and we will continue to contribute to the DOHaD field. However, sub-Saharan Africa still faces the dual burden of high rates of maternal and child undernutrition and a growing incidence of non-communicable disease risk. Improving food security, alongside understanding the nutritionally modifiable pathways that link poor nutrition in early life with disease risk across the life-course, is of fundamental importance within this critical field of global health research.
